# Probabilistic metabolite annotation using retention time prediction and meta-learned projections

**DOI:** 10.1186/s13321-022-00613-8

**Published:** 2022-06-07

**Authors:** Constantino A. García, Alberto Gil-de-la-Fuente, Coral Barbas, Abraham Otero

**Affiliations:** 1 Department of Information Technology, Escuela Politécnica Superior, Universidad San Pablo CEU, Campus Montepríncipe, Boadilla del Monte (Madrid), 28688 Spain; 2 Centre for Metabolomics and Bioanalysis (CEMBIO), Facultad de Farmacia, Universidad San Pablo CEU, Campus Montepríncipe, Boadilla del Monte (Madrid), 28688 Spain

**Keywords:** Metabolomics, Retention time, Machine learning, Bayesian methods, Deep learning

## Abstract

Retention time information is used for metabolite annotation in metabolomic experiments. But its usefulness is hindered by the availability of experimental retention time data in metabolomic databases, and by the lack of reproducibility between different chromatographic methods. Accurate prediction of retention time for a given chromatographic method would be a valuable support for metabolite annotation. We have trained state-of-the-art machine learning regressors using the 80, 038 experimental retention times from the METLIN Small Molecule Retention Tim (SMRT) dataset. The models included deep neural networks, deep kernel learning, several gradient boosting models, and a blending approach. 5, 666 molecular descriptors and 2, 214 fingerprints (MACCS166, Extended Connectivity, and Path Fingerprints fingerprints) were generated with the alvaDesc software. The models were trained using only the descriptors, only the fingerprints, and both types of features simultaneously. Bayesian hyperparameter search was used for parameter tuning. To avoid data-leakage when reporting the performance metrics, nested cross-validation was employed. The best results were obtained by a heavily regularized deep neural network trained with cosine annealing warm restarts and stochastic weight averaging, achieving a mean and median absolute errors of $39.2 \pm 1.2\; s$ and $17.2 \pm 0.9\;s$, respectively. To the best of our knowledge, these are the most accurate predictions published up to date over the SMRT dataset. To project retention times between chromatographic methods, a novel Bayesian meta-learning approach that can learn from just a few molecules is proposed. By applying this projection between the deep neural network retention time predictions and a given chromatographic method, our approach can be integrated into a metabolite annotation workflow to obtain z-scores for the candidate annotations. To this end, it is enough that just as few as 10 molecules of a given experiment have been identified (probably by using pure metabolite standards). The use of z-scores permits considering the uncertainty in the projection when ranking candidates, and not only the accuracy. In this scenario, our results show that in 68% of the cases the correct molecule was among the top three candidates filtered by mass and ranked according to z-scores. This shows the usefulness of this information to support metabolite annotation. Python code is available on GitHub at https://github.com/constantino-garcia/cmmrt.

**Supplementary Information:**

The online version contains supplementary material available at 10.1186/s13321-022-00613-8.

## Introduction

Metabolite annotation remains the main bottleneck in untargeted metabolomics [1, 2], with the vast majority of metabolites being left as unidentified [3]. Beyond the molecule’s mass, other molecule’s properties such as Retention Time (RT), collision cross section, or the fragmentation spectrum can be very valuable during the metabolite annotation process [4, 5]. The most common approach to annotate metabolites is to query a metabolomics database for compounds that have a mass compatible with the experimental masses. Often this query returns multiple annotation candidates for the same mass. Next, the researcher tries to discard, or score according to plausibility, the candidate annotations using other molecule’s properties [1].

Liquid Chromatography Mass Spectrometry (LCMS) remains the most common platform used in untargeted metabolomics. In addition to *m/z* ratio, it provides information about the Retention Time (RT), the time at which metabolites elute from the chromatographic column. By using hyphenated setups (MS/MS), the fragmentation spectra of the molecules may be obtained [6]. These spectra are very useful for ruling out candidate annotations, and they are necessary to achieve the highest confidence levels of the Metabolomics Society in metabolite annotation (levels 0 and 1 [7]). However, obtaining them requires hyphenated setups which are more expensive and complex. Even when this type of instrumentation is used, the fragmentation spectrum of every molecule of interest is not always available due to instrumentation limitations or time constraints for the analysis. Also, sometimes the amount of sample available is not sufficient for MS/MS analysis. Hence, especially in pilot untargeted studies where unambiguous identification is not crucial (that is, where confidence level 2 of the Metabolomics Society is enough), often the fragmentation spectra are not available, and the annotation has to be done with just *m/z* ratios and RTs.

Obtaining molecule’s properties experimentally (such as the Retention Time (RT) or the fragmentation spectrum) requires the analysis of pure standards, which is a long, tedious and expensive process. Therefore, metabolomic databases often lack this information, especially for new metabolites that are still being discovered. Furthermore, the variability of the experimental setups means that different values are often obtained for these features in different setups [6, 8]. The reliable prediction of these features from the structures of molecules using machine learning techniques is therefore a compelling alternative to their experimental generation [9–12].

Computational prediction of the Retention Time (RT) has been shown to be useful for molecule annotation in proteomics [13, 14] and lipidomics [15, 16]. However, until recently the prediction of small molecules Retention Time (RT) remained a challenge due to the small size (usually a few hundreds) of the publicly available Retention Time (RT) datasets [17]. This size prevented the training of machine learning models capable of accurately predicting the Retention Time (RT) of the large variety of small molecules involved in the typical metabolomic study, being the efforts in this direction limited to the prediction of the Retention Time (RT) of some concrete type of small molecules [14, 16, 18], or of the order of elution of the molecules [19, 20]. This situation changed recently with the publication of more than 80,000 experimental RTs collected through reversed-phase Liquid Chromatography Mass Spectrometry (LCMS) from the METLIN Small Molecule Retention Time (SMRT) dataset [21], which has renewed interest in the Retention Time (RT) prediction of small molecules [17, 22–26].

In this paper we have tested the performance of several state-of-the-art machine learning models for the task of Retention Time (RT) prediction using the SMRT dataset. In the evaluation presented in [21] the molecules that were not retained by the column were excluded. In our evaluation, both retained and non-retained molecules will be considered. Non-retained molecules are typically ignored in metabolomics experiments. However, the ultimate goal of the machine learning model would be the computational prediction of the RTs of a set of molecules present in a metabolomics database based on their chemical structures, to confirm or discard candidate metabolite annotations. In this scenario, it is unknown in advance whether a molecule of the database is going to be retained or not, and therefore it is desirable to predict as accurately as possible the RTs of the non-retained molecules.

Hyperparameter search for the models was performed with the Tree-structured Parzen Estimator (TPE) algorithm [27], and a nested cross-validation was used in the evaluation. The best model was a Deep Neural Network (DNN) trained using molecular fingerprints, which improved the performance of the best previous models to predict Retention Time (RT) [24, 26].

**Fig. 1:**
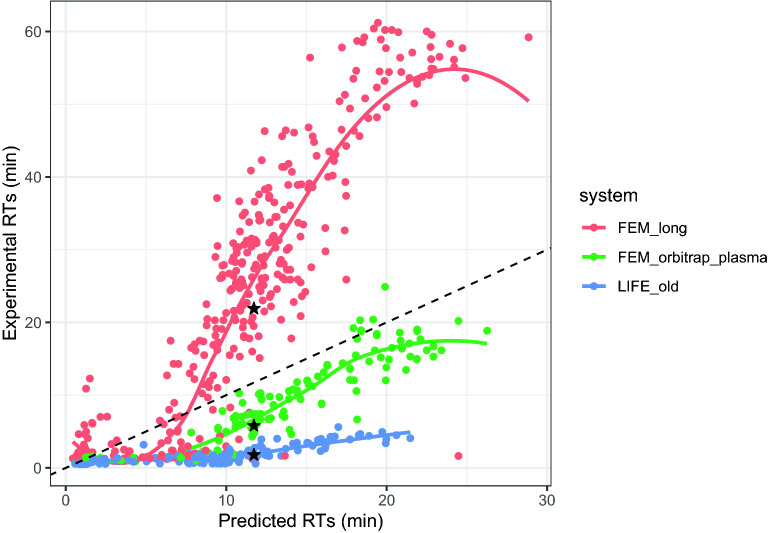
RTs measured in different CMs (y-axis) compared with the predictions of a machine learning model trained on SMRT (x-axis). The figure also shows the RTs of the same molecule (PubChem ID 10742) in the different CMs (shown with the star shapes), which illustrates the variability in the times measured with different experimental configurations. Since the model has been trained on a single Chromatographic Method (CM), the experimental RTs on different CMs do not match its predictions (dashed line). This figure illustrates the need for a projection method able to *translate* the predictions of a model trained on a specific Chromatographic Method (CM) to different CMs

Having a machine learning model capable of accurately predicting the Retention Time (RT) would enable filtering out annotations with similar mass but different RTs. However, note that a model trained on the SMRT dataset can only accurately predict RTs for a Chromatographic Method (CM) identical to the one employed to collect this data. Since laboratories usually customize the Chromatographic Method (CM) for the needs of each experiment, a SMRT-based model cannot be directly applied to experimental data from other laboratories, or even other experiments conducted in the same laboratory. However, if CMs are similar, elution order is largely preserved [8], which enables the construction of a projection function that maps RTs in one Chromatographic Method (CM) to RTs in another Chromatographic Method (CM). Figure 1 illustrates both the dependency of the RTs with the Chromatographic Method (CM), and the conservation of the elution order. To build such a projection function, a set of known molecules whose RTs is known in both CMs is needed.

**Fig. 2:**
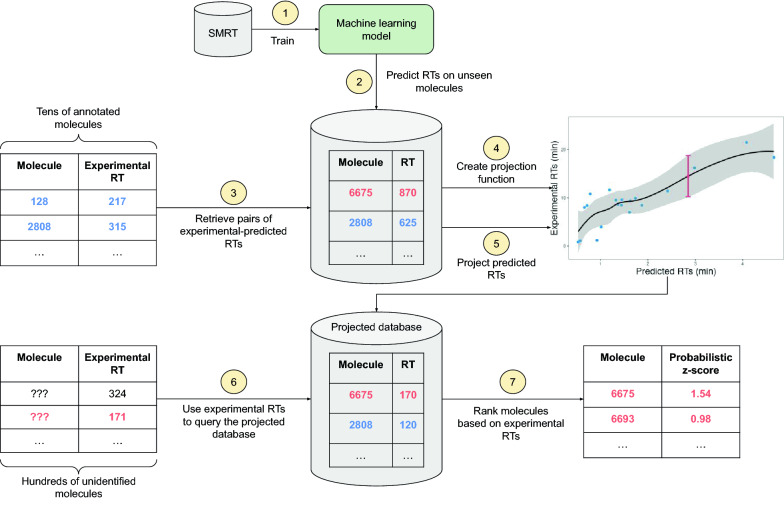
Illustrative workflow to exploit a machine learning model trained on a large dataset (here, SMRT) to annotate metabolites. Steps 1-2: a RTs database is created using a predictive model trained on the SMRT dataset. Step 3: to use this database, a researcher provides the experimental RTs of a few molecules whose identity is known. The molecule identities are then used to retrieve the corresponding predicted RTs from the database to create pairs of experimental-predicted RTs. Step 4: a projection function mapping predicted RTs to experimental RTs is learned from these pairs. Step 5: the researcher then provides experimental *m/z* (not shown in the figure) of the molecules he/she is trying to identify. Molecules are filtered using the *m/z* ratio and the predicted RTs of those molecules are then projected to experimental RTs to create a “projected database”. Step 6: the researcher finally uses the experimental RTs to query the projected database. Step 7: the results retrieved from it would enable scoring candidates with similar *m/z* but different RTs

Figure 2 shows a possible workflow to exploit the Retention Time (RT) predictions of a machine learning model and a projection method during the metabolite annotation process. Although not explicitly shown in the figure, we assume that RTs are used in conjunction with the *m/z* ratio. In the center of Fig. 2 there is a large database containing molecule identities and their main chemical properties, including RTs. The RTs stored in the database are computed using the predictive model trained on the SMRT dataset (step 1). The creation of a database (step 2) avoids running complex predictive models in real-time. Also, note that this database may include molecules not observed in the SMRT dataset. To use this database, a researcher provides the experimental RTs (as measured in his/her Chromatographic Method (CM)) of a few molecules whose identity is known (step 3). These molecules will typically be pure metabolite standards added to the sample. The molecule identities are then used to retrieve the corresponding predicted RTs from the database, which will be subsequently used to create pairs of experimental-predicted RTs (step 3). A projection function mapping predicted RTs to experimental RTs is learned from these pairs (step 4). The researcher then provides experimental *m/z* (not shown in the figure to avoid clutter) of the molecules he/she is trying to identify. The *m/z* ratios are used as a first filter to obtain candidate annotations from the database. The predicted RTs of the filtered molecules are then projected to experimental RTs to create a “projected database” (step 5). Note that the projected database is much smaller than the original one due to the *m/z* filtering, which makes the projection computationally efficient. The researcher finally uses the experimental RTs to query the projected database (step 6). The results retrieved from it would enable scoring candidates with similar *m/z* but different RTs (step 7).

To make this workflow practical, it is desirable that the projection function can be learned from a very small dataset, so that the researcher only has to identify a small set of molecules. To that end, this work proposes a Bayesian meta-learning approach to project the predicted RTs to a specific Chromatographic Method (CM) based on just a few identified molecules. This approach has the advantage of being able to generalize from a small training set while providing confidence intervals for the Retention Time (RT) projections between CMs, and not only a point estimate. We demonstrate the ability of the proposed projection method to learn from few samples by testing not only its predictive accuracy, but also its ability to rank the correct metabolite identity among the top three candidates based on their RTs.

## Materials

The METLIN Small Molecule Retention Time (SMRT) dataset consists of the experimental retention times of 80,038 small molecules from the METLIN library [21]. All RTs were obtained using reverse-phase chromatography with high-performance liquid chromatography-mass spectrometry (HPLC-MS). The dataset has a wide variety of small molecules analysed under the same conditions, including metabolites, natural products and drug-like small molecules. It also includes non-retained molecules; these are compounds that are not retained in the column and elute before gradient starts, typically within the first minute. Hence, RTs of the non-retained molecules are considerably smaller than RTs of the retained molecules. Although some authors ignore the non-retained molecules when validating machine learning models, the whole dataset was used for both training and validating the regressors of this paper. The rationale for this is that these machine learning models are going to be used to predict RTs of metabolites in a database (see Fig. 2). Then, these predictions could be used to filter and rank experimental data. If a regressor is trained without non-retained RTs it will only be able to predict retained RTs, even for a non-retained metabolite *A* in the database. If in an experiment there is an unidentified metabolite *B* with a similar *m/z* and whose RT is close to *A*’s (wrongly) predicted Retention Time (RT), the system will propose metabolite *A* as a candidate annotation for *B*. Hence the interest in training the regressors with both retained and non-retained molecules.

The SMRT dataset has been made public including the PubChem numbers and SDF files representing their chemical structure [28], together with their experimental RT information. In this work, these chemical structures were used to obtain a wide variety of features describing relevant properties of the molecules. These features were computed using alvaDesc [29, 30] and include both fingerprints and molecular descriptors. Specifically, alvaDesc permits the computation of MACCS166 fingerprints, Extended Connectivity Fingerprints (ECFP) [31] and Path Fingerprints (PFP), making a total of 2, 214 fingerprints. Additionally, the 5, 666 molecular descriptors supported by alvaDesc were also generated; the complete list can be seen in [32]. All the descriptors and fingerprints obtained with alvaDesc were used to feed the regressors.

Following [21], we also used the PredRet database [8] for validating the projections from predicted to experimental RTs. The PredRet is a database of experimental RTs from different chromatographic systems commonly used for building and testing projection models between pairs of CMs.

## Methods

First we shall describe the different machine learning models used to predict the RTs, and then we shall present our Bayesian approach to project the RTs to a given Chromatographic Method (CM).

### Prediction of retention times with machine learning

Several state of the art machine learning regressors were tested for predicting the RTs using three different sets of features: fingerprints only, descriptors only and fingerprints+descriptors. Parameter search was used for tuning all models with the exception of CatBoost-based regressors (see "Gradient boosting" Section for the rationale). We have also created an ensemble with all the trained models to attempt to further improve Retention Time (RT) prediction [33]. Some of the choices for the regressors can be understood by the need of having diversity in their predictions to increase the chances of the ensemble improving their individual performances (see "Blending" Section).

#### Preprocessing of descriptors and fingerprints

Descriptors were first standardized and imputed using median imputation when alvaDesc was not able to generate some descriptor. If imputation was needed, a missing indicator was added, enabling the regressors to account for missingness despite the imputation. Features with 0 variance were removed. Highly correlated features were also eliminated (correlation $> 0.9$); this conservative threshold was not tuned since all tested regressors are robust against collinearity. The main benefit of removing correlated features is memory saving.

The only preprocessing applied to the fingerprint features was removing those with low variance. Treating each feature *X* as a binary Bernouilli random variable, the variance threshold was selected using $\text {Var}[X]=p (1-p)$, were *p* is a parameter to be tuned (see "Bayesian hyperparameter search" Section) which is usually set to a high value (typically $>0.9$).

**Fig. 3:**
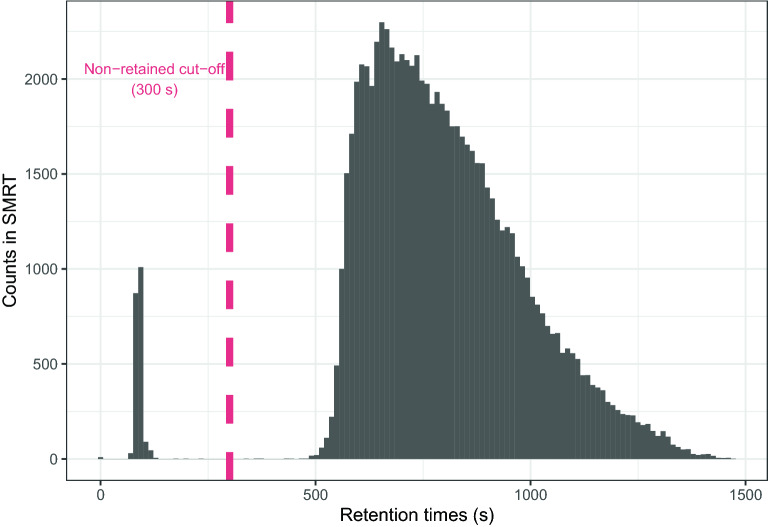
Histogram of the RTs in the SMRT dataset. The distribution is clearly bimodal due to the presence of non-retained molecules. In this paper, a molecule is considered as non-retained if its Retention Time (RT) is smaller than 300s

Taking inspiration from [24], an additional binary feature was added to each molecule representation indicating whether the molecule is retained or not. Since in a real world application this information would not be available, this feature must also be predicted. To that end, we trained a eXtreme Gradient Boosting (XGBoost) classifier. As suggested by Fig. 3, a molecule was considered non-retained if its Retention Time (RT) was smaller than 5 minutes. The XGBoost classifier was tuned using the same procedure described in "Bayesian hyperparameter search" Section for the regressors, although the metric to be maximized in this case was the F1 score. Preliminary results suggested that using fingerprints, descriptors, and fingerprints+descriptors yielded similar results, so we used only fingerprints as features for speed.

#### Gradient boosting

Gradient Boosting Machines (GBMs) have already been considered in state of the art methods for Retention Time (RT) prediction [24]. In this work, several GBMs were tested, using slightly different approaches for the hyperparameter search. In addition to the interest of comparing several GBMs, the use of different combinations of GBMs and tuning options was partially motivated by the need of having diversity in the predictions for building a good ensemble (see "Blending" Section). Specifically, we tested:

- XGBoost [34]: it is probably the most-commonly used GBM, and it was employed for Retention Time (RT) prediction in [24]. Bayesian search was applied on different regressors fed using fingerprints, descriptors and fingerprints+descriptors. Among the tuned parameters, the most relevant ones include the number of boosting rounds, the maximum depth of the trees, subsampling parameters (either by column, by tree or by level), regularization parameters (such as $L_1$ and $L_2$ regularization) and parameters controlling the conservativeness of the algorithm (usually referred as $\gamma$ and *minimum child weight*).
- Gradient Boosting Machine (lightGBM) [35]: it is a well-known alternative to XGBoost, with optimizations for speed and memory usage (hence, its name). Furthermore, stepwise optimization methods particularly designed for lightGBM can be used. This avoids the need for Bayesian search, further reducing tuning times by exploiting heuristics. The hyperparameters were selected in the following order: $L_1$ regularization, $L_2$ regularization, maximum number of leaves, proportion of randomly selected features on each tree, bagging fraction, bagging frequency (it controls the number of iterations between bagging) and the minimum number of samples in the leaves.
- CatBoost [36]: an interesting question regarding the non-retained molecules is if their inclusion as training data improves the performance of the regressors. To investigate this question, the performance of a regressor when trained with different weights for the retained and non-retained molecules can be evaluated. Different weights for both types of molecules can also help with the unbalance between retained and non-retained molecules. Since the ratio of non-retained to retained molecules is approximately 1/40 in the SMRT dataset, the weight of the retained molecules was set to 1, whereas the weight of the non-retained molecules was varied between $10^{-6}$ (effectively ignoring them) and 80 (hence the global influence of the non-retained molecules is approximately twice the influence of the retained ones). However, using the same approach as with previous regressors would require a full Bayesian search for each weight of the non-retained molecules. Instead of tuning parameters for each weight, we looked for a regressor able to provide good performance with its default parameters. CatBoost was selected for this reason [36]. Note that CatBoost regressors not only permit studying the influence of the non-retained molecules in the predictions, but they also provide a useful *context* that may enable the meta-regressor of the ensemble to distinguish between retained and non-retained molecules (see "Blending" Section).

#### Deep neural network

Together with GBMs, DNNs usually achieve the best results in machine learning competitions [37, 38]. DNNs were used for Retention Time (RT) prediction in [21], where a DNN with 4 layers and regularization was proposed. Regularization is key for achieving good generalization, since even a small shallow neural network can overfit the SMRT dataset in a few epochs. Driven by this observation, we used a DNN with just 3 layers, regularized using large dropout rates. The sizes of the hidden layers, the dropout rates and the non-linear activations were determined using Bayesian hyperparameter search.

To improve the generalization ability of the DNN and to accelerate its training, we used cosine annealing warm restarts [39]. The number of restarts and the length of the cosine annealing were also subject to hyperparameter search. After the training with warm restarts, we employed Stochastic Weight Averaging (SWA) using a constant learning rate schedule. With this setting, SWA just consists of training the DNN for a few extra epochs (whose optimal value is to be determined during hyperparameter search), and then averaging the weights of the DNN along the trajectory followed during optimization. In [40], the authors suggest that SWA leads to wider minima, which is hypothesized to result in better generalization than conventionally trained DNN.

Finally, quantile transformation was applied to RTs before fitting. The method transforms RTs to follow a standardized normal distribution. This may facilitate learning since the last layer does not need to learn large weights to match the untransformed RTs.

#### Kernel methods

Support Vector Machines (SVMs) have already been considered for a wide variety of applications related to metabolites, including elution order prediction [19]. Although we tested SVMs with both descriptors and fingerprints, performance of both regressors was poor. As an alternative to this classic kernel method, we considered Deep Kernel Learning (DKL) [41]. DKL can be interpreted as a DNN whose last layer has been substituted by a Gaussian Process (GP). This permits leveraging both the ability of deep learning for extracting relevant features from the raw-inputs, and the non-parametric flexibility of GPs. The combination of the DNN and the GP kernel can also be viewed as a new flexible kernel which can be used as a drop-in replacement for standard kernels. DKLs were tested using fingerprints, descriptors and fingerprints+descriptors.

Following the observations from "Deep neural network" Section, we employed a highly regularized DNN. Besides dropout, we also considered batch-normalization [42], not only because of its regularization capabilities, but also because it keeps activations from the network within a predictable range. This eases the use of kernel interpolation (specifically, KISS-GP  [43]) to approximate the GP kernel, which enables fast computations. Quantile transformation was also applied to the RTs.

DKL was trained using early stopping, and the learning rate was tuned during parameter search. Similar to the DNNs from "Deep neural network" Section, the specific architecture and regularization were subject to parameter search. Learning rate scheduling was used, reducing the learning rate when validation loss was stacked in a plateau. The *patience* argument before decreasing the learning rate was also tuned. Finally, three kernels were considered during hyperparameter tuning: the squared exponential kernel, the linear kernel and a spectral mixture kernel with four components [44]. A full list of the mathematical expressions of the kernels used in this paper can be found in Section S3 of Additional file 1.

#### Blending

We tested if the combination of the different regressors could improve their individual predictions. We used blending [45] to build a meta-regressor which learns to combine the predictions of the so-called base-regressors. Blending is a popular alternative to stacked generalization (or stacking) [46] which has lower computational demand and it is simpler, resulting in less likelihood of information leakage. With large datasets like SMRT, blending and stacking usually yield similar results. Hence, since the meta-regressor is also subject to parameter tuning, blending was used for faster training.

To train a meta-regressor with blending, a holdout set is created using a small subset of the training set. In our experiments, we used a 80-20% split. The base-regressors are trained on the 80% of the data, and their predictions for the holdout dataset are stored. The meta-regressor then learns to combine the predictions of the base-regressors using the predictions on the holdout dataset. Note that an instance on the original training data is only used just once for training, either on the base-regressors or in the meta-regressor, avoiding information leakage. This procedure for training the meta-regressor is also outlined in Fig. S1 of Additional file 1.

A random forest was used as meta-regressor, tuning its main parameters through Bayesian optimization. The parameters tuned were the number of trees, the maximum depth of each tree, the maximum number of features considered at each split, the minimum number of samples before considering a split and the minimum number of samples at a leaf.

#### Bayesian hyperparameter search

Most regressors with the exception of lightGBMs (tuned using iterative search for speed tuning) and CatBoosters (not tuned due to its good default values) were tuned using Bayesian hyperparameter search. The *p* parameter controlling the thresholding of binary features was also optimized (see "Preprocessing of descriptors and fingerprints" Section). The parameters were tested following the predictions of a TPE algorithm [27]. The TPE algorithm works by suggesting the parameters that maximize the expected improvement in the score being maximized, which in this paper was the negative of the MEDian Absolute Error (MEDAE). This permits balancing exploration versus explotation, obtaining a set of hyperparameters with good performance in fewer iterations than other approaches like grid search. In our experiments each model performed 50 iterations of the Bayesian search.

Regarding the optimization of the blended regressor, it should be noted that it proceeds greedily. That is, base-regressors are tuned individually, and the predictions of the best performing parameters are then used to create the training set for the meta-regressor. Finally, the parameters of the latter are optimized. It may be argued that this approach is suboptimal, since the base-regressors cannot be tuned to complement each other. However, jointly optimizing all base-regressors and the meta-regressor is difficult due to the dimensionality of the search space. Furthermore, this approach would not permit drawing conclusions from the performance of the base-regressors, which is part of the objectives of this work.

#### Validation procedure

To avoid data-leakage when reporting the performance of the different models, nested stratified cross-validation was used. Nested cross-validation guarantees that different data is used to tune model parameters and to evaluate its performance by means of outer and inner cross-validation loops [47]. In the outer loop, train/test splits are generated, which are then used for averaging the test scores over several data splits. In the inner loop, the train set is further split in train/validation subsets. The best parameters are selected by minimizing the MEDAE on the validation splits. We used 5-folds and 7-folds stratified cross-validations in the outer and inner loops, respectively. To ensure that the distribution of RTs is representative of the population in all folds, stratification was performed by separating the target variable (RTs) into 6 different bins. The validation procedure is also summarized in Fig. S1 of Additional file 1.

The Bayesian hyperparameter search ("Bayesian hyperparameter search" Section) and the validation procedure described in this section approximately required 2.5 months of computational time in a computer with an AMD Ryzen Threadripper 2970WX with 24 cores at 1.85 Gz, and a NVIDIA GeForce RTX 2080 GPU.

### Projection between chromatographic methods

Machine learning models trained on a given Retention Time (RT) dataset (SMRT in this work) cannot be directly used to predict experimental RTs from other Chromatographic Method (CM)s due to the variability of the experimental setups. To exploit the knowledge of a predictive model trained on the SMRT, a second model projecting the predicted RTs to the specific Chromatographic Method (CM) used in an experiment is needed.

Given a specific Chromatographic Method (CM), the projection function can be learned if some of the experimental metabolites have been identified, and therefore both their experimental and predicted RTs are known (step 3 in Fig. 2). For the workflow in Fig. 2 to be practical, it is desirable that the projection function can be learned from a small dataset (tens of molecules) so that the researcher has to identify just a few molecules. In practice, this would probably be accomplished by adding pure metabolite standards to the sample. The more standards that need to be used, the more time and money will be required. Hence the interest in minimizing their number.

Bayesian methods are particularly well suited to solve classification/regression problems when data is scarce. This is due to their ability to incorporate prior knowledge about the problem. If the prior provides useful inductive biases for the task at hand, only a few samples may be needed to learn a proper solution to the problem [48]. Hence, under the Bayesian paradigm, the issue of learning from few data becomes how to specify a suitable prior for the problem.

Meta-learning has recently arose as a possible solution for acquiring useful prior knowledge. In meta-learning, knowledge is gained by solving a set of tasks (meta-tasks), which is then exploited to solve a closely-related but different task (target-task). In the Bayesian setting, meta-tasks are used to learn a useful prior distribution, which is then used as starting point to solve the target-task. This is done by incorporating new evidence provided by the target-task into the prior, which results in the so-called posterior distribution.

**Fig. 4:**
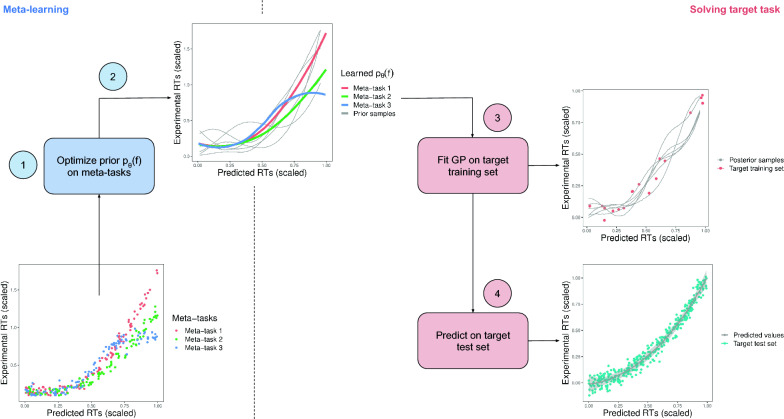
Overview of the meta-learning approach to RTs projection. 1) The meta-tasks consist of creating projection functions mapping predicted RTs to experimental RTs in several Chromatographic Method (CM)s. Each target Chromatographic Method (CM) is a different meta-task. 2) During meta-learning a prior distribution $p_{\theta }(f)$ on the projection functions is learned. This prior contains the learned projection functions in the meta-tasks (shown in color), and also any other function with similar properties to those observed in the dataset (shown in gray). 3) To solve a target-task, the prior distribution $p_{\theta }(f)$ is updated with new evidence provided by the target training set, resulting in the so-called posterior distribution. 4) The posterior distribution is used to evaluate the performance on a target test set

Hence, we propose the use of meta-learning to solve the problem of learning from few samples. The outline of the approach is shown in Fig. 4. We shall consider that the set of meta-tasks $\mathcal {M}$ consists of *m* datasets $\mathcal {M}=\{\mathcal {D}_i\}_{i=1}^m$, each corresponding to a different Chromatographic Method (CM). Each dataset $\mathcal {D}_i$ contains predicted RTs $\mathbf {x}^i$, as well as the experimental RTs obtained with a specific Chromatographic Method (CM), $\varvec{y}^i$. Hence, $\mathcal {D}_i=\{\mathbf {x}^i, \varvec{y}^i\}$ is a single meta-task and we would like to map $\varvec{x}^i$ to $\varvec{y}^i$ using a smooth function $f(\cdot )$. The predicted RTs are obtained by using the best predictive model from "Prediction of retention times with machine learning" Section. In our problem, meta-tasks are used to learn a prior distribution *p*(*f*) over the functions $f(\cdot )$ translating predicted RTs to experimental RTs of different CMs. Let us consider that we have gathered experimental RTs using the CMs *A*, *B* and *C*. During meta-learning, the projection functions

$$\begin{aligned} f_A(\varvec{x}^A) \approx \varvec{y}^A, f_B(\varvec{x}^B) \approx \varvec{y}^B, \text { and } f_C(\varvec{x}^C) \approx \varvec{y}^C, \end{aligned}$$

will be constructed. These functions should be considered as samples drawn from the same distribution *p*(*f*). The aim of meta-learning is to learn a plausible prior *p*(*f*) that explains all observed samples $f_A(\cdot ), f_B(\cdot )$ and $f_C(\cdot )$.

In addition to the the meta-tasks we have the target-task $\widetilde{\mathcal {D}}=\{\varvec{\tilde{x}}, \varvec{\tilde{y}}, \varvec{\tilde{x}^*}, \varvec{\tilde{y}^*}\}$. Again, a single target-task is comprised of data from a single Chromatographic Method (CM). Intuitively, the target training points $\{\varvec{\tilde{x}}, \varvec{\tilde{y}}\}$ represent molecules whose identity is known (step 3 in Fig. 2) whereas the target test points $\{\varvec{\tilde{x}^*},\varvec{\tilde{y}^*}\}$ are molecules whose identity is to be discovered (step 6 in Fig. 2). We assume that the number of annotated molecules is small (indeed, this is the main difference between a meta-task and a target-task). Note that, when solving the target-task, the prior distribution *p*(*f*) learned during meta-learning is updated with the new evidence $\{\varvec{\tilde{x}}, \varvec{\tilde{y}}\}$, which should enable the prediction/ranking of $\{\varvec{\tilde{x}^*},\varvec{\tilde{y}^*}\}$.

GPs are particularly suited as projection model: they represent a distribution over functions, they can perform regression on smalls amount of data, and they can incorporate prior knowledge using the Bayesian framework. Hence, we shall consider:

$$\begin{aligned}&f(\cdot ) \sim \mathcal {GP}\left( m_{\varvec{\theta }_m}(\cdot ), k_{\varvec{\theta }_k}(\cdot , \cdot )\right) \text { or equivalently}\\&p_{\varvec{\theta }}(f)= \mathcal {GP}\left( f \mid m_{\varvec{\theta }_m}, k_{\varvec{\theta }_k}\right) ,\qquad \varvec{\theta }=[\varvec{\theta }_m, \varvec{\theta }_k] \end{aligned}$$

where the mean and kernel functions of the GP are parametrized with $\varvec{\theta }_m$ and $\varvec{\theta }_k$, respectively. Hence, the whole prior is parametrized with $\varvec{\theta }=[\varvec{\theta }_m, \varvec{\theta }_k]$. These parameters are learned by minimizing the negative Leave One Out (LOO) log predictive probability on the meta-tasks (see Algorithm 1, where $\varvec{y}^i_{-j}$ means all targets but the *j*-th item). The use of the LOO-based loss instead of the usual log marginal loss is based on the observation that cross-validation procedures (such as LOO) should be more robust against possible model misspecifications [49, Section 4.8]. Once the parameters have been learned, they can be used to specify a prior that is expected to generalize well on the target-task. Indeed, to avoid overfitting, $\varvec{\theta }$ is not optimized while solving the target-tasks. The only parameter estimated with target-task data is the variance of the residuals. This is done by maximizing type II maximum likelihood during 250 epochs with an Adam optimizer with learning rate set to 0.01.
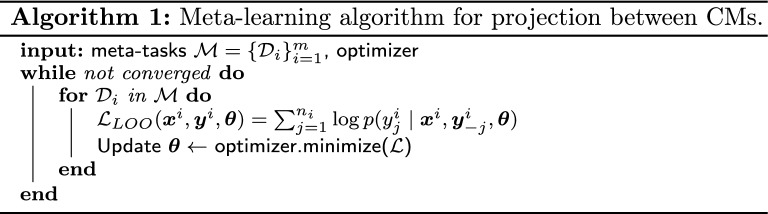


It is worth noting that the proposed meta-learning method fits well the Retention Time (RT)-based filtering workflow shown in Fig. 2. In this scenario, a query from a researcher corresponds to a target-task, which exploits the information provided by a previously meta-learned prior. Furthermore, although computing new predictions with the projection function scales quadratically with the training data, it will typically train on tens of RTs. Hence, it would only take tenths of a second to map experimental RTs to predicted RTs. Next sections present the data preprocessing and the parameter ($m_{\varvec{\theta }_m}$, $k_{\varvec{\theta }_k}$) selection process used to devise our projection method.

#### Experimental setup and data preprocessing

In [21] the non-retained molecules were ignored for validating the projection method, and we adopt the same methodology here. The rationale for this is that in an experiment is easy to know if a molecule has been retained or not, and a researcher would not use a RTs database to try to annotate non-retained metabolites.

To avoid data-leakage during validation, we ensured that the meta-tasks data, target training data, and target test data did not overlap. To that end, we used a leave-one-Chromatographic Method (CM)-out approach. That is, data from a specific Chromatographic Method (CM) could only be used as either part of the meta-tasks or as the target-task. Hence, when using a Chromatographic Method (CM) as target-task, meta-learning was used on the remainder of CMs. Following [21], the following CMs were used to create the target-tasks: FEM long (342 molecules), FEM orbitrap plasma (133), LIFE old (148) and RIKEN (271). The number of molecules in the remainder of CMs is 2418.

For a specific target-Chromatographic Method (CM) (one of the four above mentioned), and after meta-learning on the meta-tasks, the target training data is created by subsampling the Chromatographic Method (CM) data (and the remainder of RTs are used as target test data). To obtain a good projection, researchers are expected to add standards spanning the whole range of the experimental RTs. To mimic this behaviour, stratified sampling was used. Sampling was repeated 10 times for each number of training points to obtain error estimates. To study the robustness of the projection method when only a few metabolites are known, the number of training points was varied between 10 and 50; 50 was the number of molecules used in [21].

All GP models share the same preprocessing steps despite their mean and kernel functions. RTs are transformed to log space using

$$\begin{aligned} \bar{\varvec{x}} = \log (1 + \varvec{x}) \qquad \text { and } \bar{\varvec{y}} = \log (1 + \varvec{y}), \end{aligned}$$

where $\varvec{x}$ and $\varvec{y}$ may belong to any meta-task $\mathcal {D}_i$ or any target-task $\widetilde{\mathcal {D}}$. The motivation for using this transformation is twofold. On one hand, RTs take only positive values. Without any transformation, the model has to learn this restriction on its own, which may be difficult in the scarce data scenario. By using the transformed RTs $\bar{\varvec{y}}$, the model learns to predict a target without any restriction. Then, the inverse transformation maps back $\bar{\varvec{y}}$ to the positive interval, forcing positiveness in the projected RTs. On the other hand, by also applying the transformation to $\varvec{x}$, the non-linear relationship between $\varvec{x}$ and $\varvec{y}$ linearizes, which could enable the use of simpler kernels.

After the log-transformation, and since the software used to implement GPs is geared towards using inputs normalized to [0, 1] and outcomes normalized to $[-1, 1]$, data is further scaled using robust statistics. We used

$$\begin{aligned} \bar{\bar{x}} = \left( \frac{\bar{x} - \text {median}(\bar{x})}{0.741\cdot \text {IQR}(\bar{x})} + 3\right) / 6 \qquad \text { and } \qquad \bar{\bar{y}} = \left( \frac{\bar{y} - \text {median}(\bar{y})}{0.741\cdot \text {IQR}(\bar{y})}\right) / 3, \end{aligned}$$

where $\text {IQR}$ denotes the interquartile range. The constant 0.741 is used because, for normal populations, the standard deviation fulfills $\sigma \approx 0.741 \cdot \text {IQR}$. Hence, under the normality assumption, 99.7% of the transformed $\bar{\bar{x}}$ will be on the [0, 1] range and 99.7% of the transformed $\bar{\bar{y}}$ will be on the $[-1, 1]$ range. Despite the different last preprocessing step for predicted ($\varvec{x}$) and experimental ($\varvec{y}$) RTs, both transformations are learned using the predicted RTs. Thus, there is no Chromatographic Method (CM)-dependent scaling.

#### Comparison of meta-learned GP models

We evaluated the performance of the different meta-learned GPs models arising from various choices of their two parameters:

- Mean function $m_{\varvec{\theta }_m}(\cdot )$: a typical parametrization of a GP when no prior information is available about the mean is $f(\cdot ) \sim \mathcal {GP}\left( 0, k_{\varvec{\theta }_k}(\cdot , \cdot )\right)$; that is, $m_{\varvec{\theta }_m}(\cdot )=0$. The underlying assumption is that all relevant prior information can be incorporated into the kernel parameters $\varvec{\theta }_k$. However, [50] shows that learning a mean function $m_{\varvec{\theta }_m}(\cdot )$ (either alone or combined with kernel learning) can outperform kernel learning alone. We tested this in our problem by studying GPs with a constant mean function, and GPs with a mean function parametrized with a neural network. In our experiments, we used a neural network with two hidden layers with 128 units and leaky-ReLU activations.
- Kernel function $k_{\varvec{\theta }_k}(\cdot , \cdot )$: different kernels result in different properties of the projection function. We compared the commonly used kernels and combinations of them. Specifically, we tested the squared exponential kernel, Matérn kernels with $\nu =1.5$ and $\nu =2.5$, the polynomial kernel of degree 4, a linear combination of two squared exponential kernels, a linear combination of a linear kernel and a squared exponential kernel, and a linear combination of a squared exponential kernel and a polynomial kernel of degree 4. A full list of the mathematical expressions for these kernels can be found in Section S1 of Additional file 1.

The experimental setup described in "Experimental setup and data preprocessing" Section is used. We focused on the performance of the models in the low-data regime using just 10 training data points. For a single target-task $\widetilde{\mathcal {D}}=\{\varvec{\tilde{x}}, \varvec{\tilde{y}}, \varvec{\tilde{x}^*}, \varvec{\tilde{y}^*}\}$, the predictive marginal log-likelihood

1 $$\begin{aligned} \mathcal {L}_{\mathcal {D}} = p(\varvec{\tilde{y}^*} \mid \mathcal {D}, \varvec{\tilde{x}}, \varvec{\tilde{y}}, \varvec{\tilde{x}^*}) = \int p \left( \varvec{\tilde{y}^*} \mid f(\varvec{\tilde{x}^*}) \right) p\left( f(\varvec{\tilde{x}^*}) \mid \mathcal {D}, \varvec{\tilde{x}}, \varvec{\tilde{y}}, \varvec{\tilde{x}}^*\right) d f. \end{aligned}$$

was used as metric of the model’s performance.

To obtain a single metric while taking into account the possible differences in the scales of the marginal log-likelihoods for the different CMs, each $\mathcal {L}_{\mathcal {D}}$ was compared with the marginal log-likelihood of a reference model $\mathcal {L}_{\mathcal {D}}^{\text {ref}}$: $\Delta \mathcal {L}_{\mathcal {D}} = \mathcal {L}_{\mathcal {D}}-\mathcal {L}_{\mathcal {D}}^{\text {ref}}$. We used as reference model a GP with constant mean and squared exponential kernel trained on the target-task without meta-learning. This model was trained by optimizing type II maximum likelihood during 500 epochs using an Adam optimizer with learning rate set to 0.01. The final metric $\Delta \mathcal {L}_{\text {avg}}$ was obtained by averaging across the four test-tasks and repetitions. Values $\Delta \mathcal {L}_{\text {avg}} > 0$ correspond with models that perform better (in average) than the reference one. Note that this not only permits the comparison of different meta-learned GP-models, but it also assesses the usefulness of the meta-learning approach.

Additional experiments studying the influence of the number of meta-tasks in the performance of the GP were also carried out. They are discussed in Section S3 of the Additional file 1.

#### Predictive performance of the projection function

We compared the best GP-model from "Comparison of meta-learned GP models" Section with monotonic Generalized Additive Models (GAMs) [8], robust polynomial regression [21] and piecewise polynomial regression [24]. Unfortunately, it is not possible to compute the predictive marginal likelihood for all these models. Hence, we evaluated the performance of the models attending to both their predictive accuracy as well as their ability to generate proper prediction intervals. To test the predictive accuracy of the meta-learning approach we computed the median relative error, Mean Absolute Error (MAE) and MEDAE for the target test set. To test the prediction intervals we used the interval score [51]

2 $$\begin{aligned} S(\varvec{\ell }, \varvec{u}, \varvec{\tilde{y}^*})&= \frac{1}{\text {len}(\varvec{\tilde{y}^*})}\sum _{i=1}^{\text {len}(\varvec{\tilde{y}^*})} S(\ell _i,u_i,\tilde{y}^*_i) \qquad \text { with }\nonumber \\ S(\ell _i,u_i,\tilde{y}^*_i)&= (u_i-\ell _i)+\frac{2}{\alpha }(\ell _i-\tilde{y}^*_i)\mathbbm {1}(\tilde{y}^*_i<\ell _i)+\frac{2}{\alpha }(\tilde{y}^*_i-u_i)\mathbbm {1}(\tilde{y}^*_i>u_i), \end{aligned}$$

where $\varvec{\ell }$ and $\varvec{u}$ are the lower and upper ends of the prediction interval generated for the test target points $\varvec{\tilde{y}^*}$, $\mathbbm {1}$ is the indicator function, and $\alpha$ is the coverage that the models are aiming for. We used $\alpha =0.95$ in all experiments. Equation (2) can be understood by noting that a proper prediction interval should reach a tradeoff between being as small as possible ($l_i$ should be close to $u_i$) and covering the observed values ($l_i \le \tilde{y}^*_i \le u_i$). The first term of Equation (2) just measures the length of the interval, while the second and third terms penalize having observed values outside the prediction interval (moreover, the further apart an observation is from the interval, the larger the penalty).

Note that the interval score has the same units as the RTs in $\varvec{\tilde{y}^*}$. To obtain an adimensional metric and facilitate the comparison of different target CMs, we define the scaled interval score as $S(\varvec{\ell },\varvec{u},\varvec{\tilde{y}^*}) / \text {median}\left( [\varvec{y^*}, \varvec{\tilde{y}^*}]\right) .$

#### Ranking annotations based on the projection function

We have tested the ability of the projection method to rank and filter candidate annotations in metabolomic experiments based on mass search and RT predictions. The test implements a similar workflow to that described in Fig. 2. We collected the Retention Time (RT) predictions of the best-performing model from "Prediction of retention times with machine learning" Section for the 6,823 molecules with KEGG number in the Human Metabolome DataBase (HMDB) [52]. This simulates the database used to rank candidate annotations in Fig. 2. We used the leave-one-Chromatographic Method (CM)-out approach described in "Experimental setup and data preprocessing" Section for meta-training and target-tasks evaluation. After learning a projection function on the target training set, we simulated queries against the HMDB database to annotate the molecules on the target test set. For each molecule in the target test set, an accurate mass search (10 ppm mass error, the same as [21]) was performed to retrieve all compatible molecules from HMDB. To mimic real experimental conditions, we simulated experimental errors in the mass measurement by adding random noise to the mass of the unknown molecule. The random noise had a normal distribution with zero mean and a standard deviation of 10/3 ppm so that $99.7\%$ of the errors were between $[-10 \text { ppm}, 10 \text { ppm}]$. Random noise below $-10$ ppm or above 10 ppm was truncated to guarantee that the mass search always returned the correct molecule as a candidate (note that the mass search is based on the noisy mass and not the real one). Then, the molecules were ranked using Retention Time (RT) information according to a z-score computed as

$$\begin{aligned} z=\frac{\mid \tilde{y}^* - \mu (\tilde{x}^*)\mid }{\sigma (\tilde{x}^*)}, \end{aligned}$$

where $\mu (\tilde{x}^*)$ and $\sigma (\tilde{x}^*)$ represent the GP’s mean and standard deviation for the predicted-experimental Retention Time (RT) pair $(\tilde{x}^*, \tilde{y}^*)$. The intuition for the usage of the z-score as ranking metric is to take into account not only the agreement between the real experimental Retention Time (RT) and the projected value, but also the uncertainty in the projection. We focused on mass queries returning more than three candidates and computed the percentage of results where the true molecule was ranked among the top three candidates after z-scoring. To facilitate the interpretation of the results, a baseline performance for metabolite annotation when using only mass information was also computed. In this case, if several candidates with the same mass were returned, ties were randomly broken.

## Results

### Retention time prediction with machine learning

**Fig. 5:**
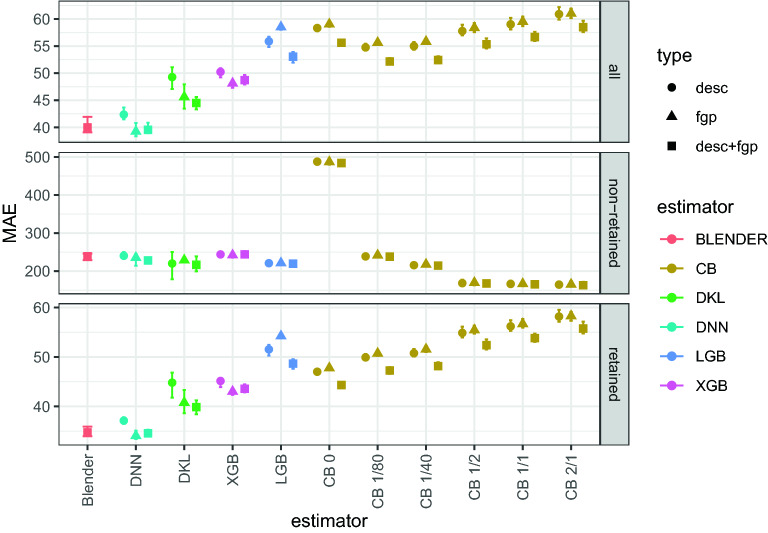
MAE results in seconds. The different estimators have been grouped by families, which are also highlighted with different colors, and approximately sorted by performance. The different panels indicate which molecules where considered for evaluating the performance: *all* includes all molecules, *non-retained* includes only those molecules considered as non-retained (Retention Time (RT) smaller than 5 minutes), and *retained* includes only retained molecules. The different shapes indicate the features used for feeding the regressors (in the legend, *fgp* and *desc* indicate fingerprints and descriptors, respectively). In the case of the blender, the features represented are descriptors+fingerprints since it is using all predictions from the base-regressors (i.e, it is using predictions from regressors using fingerprints, regressors using descriptors, and regressors using both). Error bars correspond to the 99% confidence interval of the MAE. In the figure, XGBoost, lightGBM and CatBoost have been shortened to XGB, LGB and CB, respectively. The numbers near the CB regressors correspond to the total contribution of non-retained molecules compared to retained molecules during training. For example, assigning a weight of 80 to each non-retained molecule (see "Gradient boosting" Section) results in these molecules influencing the loss function 80 times more than the retained ones. Since the relative abundance is 1/40, this leads to non-retained molecules having twice the influence of retained molecules during learning, which is shown as *CB 2/1*

MAE results for all tested regressors are summarized in Fig. 5. The MEDAE results are qualitatively similar to MAE ones, and can be found in Fig. S3 of Additional file 1. Both MAE and MEDAE are also reported in Tables 1, 2 and 3. Figure 5 shows that the DNN models outperform the other models, with the exception of the blender, which has similar results. Specifically, the DNN trained with fingerprints achieves a MEDAE of $17.2 \pm 0.9\;s$ and a MAE of $39.2 \pm 1.2\; s$ when considering all molecules, and a MEDAE of $17.2 \pm 0.9\;s$ and a MAE of $34.0 \pm 0.9\; s$ when considering retained molecules only. To the best of our knowledge, the previous top performing models achieved a MAE of $45.6 \pm 0.4\;s$ for all molecules [24], and $39.87\;s$ when only using retained molecules [26].

**Table 1:** MAE and MEDAE results for the top 4 performing regressors (mean ± standard error)

		Blender	DNN			DKL			XGB		
Features	Metric	desc+fgp	desc	fgp	desc+fgp	desc	fgp	desc+fgp	desc	fgp	desc+fgp
All	MAE (s)	$39.98\pm 1.49$	$42.35\pm 1.13$	$39.23\pm 1.2$	$39.54\pm 0.97$	$49.27\pm 2.09$	$45.60\pm 2.37$	$44.49\pm 1.11$	$50.24\pm 0.99$	$48.13\pm 0.63$	$48.72\pm 0.90$
	MEDAE (s)	$18.63\pm 1.24$	$20.02\pm 0.73$	$17.22\pm 0.89$	$18.00\pm 0.44$	$26.73\pm 2.00$	$23.25\pm 1.87$	$22.51\pm 0.73$	$27.18\pm 0.71$	$25.64\pm 0.56$	$25.32\pm 0.98$
Non-retained	MAE (s)	$239.02\pm 8.68$	$240.30\pm 8.61$	$235.46\pm 15.85$	$228.11\pm 5.41$	$220.07\pm 38.77$	$228.94\pm 6.10$	$216.61\pm 21.06$	$243.97\pm 7.14$	$242.19\pm 6.57$	$244.17\pm 7.89$
	MEDAE (s)	$87.18\pm 137.45$	$106.45\pm 180.01$	$25.10\pm 6.89$	$17.58\pm 3.80$	$128.53\pm 167.60$	$15.18\pm 5.86$	$33.40\pm 44.41$	$129.74\pm 117.80$	$134.06\pm 108.68$	$126.17\pm 97.29$
Retained	MAE (s)	$34.73\pm 1.14$	$37.11\pm 0.67$	$34.06\pm 0.86$	$34.56\pm 0.67$	$44.80\pm 2.57$	$40.77\pm 2.39$	$39.85\pm 1.50$	$45.14\pm 0.93$	$43.01\pm 0.65$	$43.57\pm 0.94$
	MEDAE (s)	$18.42\pm 1.37$	$19.99\pm 0.72$	$17.21\pm 0.87$	$18.01\pm 0.43$	$26.79\pm 1.99$	$23.29\pm 1.86$	$22.58\pm 0.76$	$26.90\pm 0.58$	$25.35\pm 0.56$	$25.04\pm 0.79$

In this table, *desc*, *fgp* and *desc+fgp* refer to the input features used by each regressor. *desc* means descriptors, *fgp* means fingerprints and *desc+fgp* means that both descriptors and fingerprints have been used. Note that some standard errors are larger than the mean MAE/MEDAE due to the presence of outliers. See Figs. 5 and S3 (the latter in Additional file 1) for better error estimates

**Table 2:** MAE and MEDAE results for LightGBM and weighted CatBoost regressors with small overall weights for non-retained molecules (0, 1/80 and 1/40)

		LGB			CB 0			CB 1/80			CB 1/40		
Features	Metric	desc	fgp	desc+fgp	desc	fgp	desc+fgp	desc	fgp	desc+fgp	desc	fgp	desc+fgp
All	MAE (s)	$55.88\pm 1.03$	$58.51\pm 0.42$	$53.04\pm 1.01$	$58.33\pm 0.52$	$59.05\pm 0.54$	$55.62\pm 0.44$	$54.76\pm 0.62$	$55.64\pm 0.44$	$52.16\pm 0.59$	$54.99\pm 0.70$	$55.83\pm 0.49$	$52.42\pm 0.67$
	MEDAE (s)	$32.92\pm 0.86$	$35.61\pm 0.77$	$30.60\pm 0.86$	$32.24\pm 0.27$	$32.95\pm 0.25$	$29.99\pm 0.20$	$32.34\pm 0.38$	$33.21\pm 0.43$	$30.04\pm 0.32$	$32.39\pm 0.10$	$33.42\pm 0.30$	$30.25\pm 0.38$
Non-retained	MAE (s)	$220.95\pm 3.37$	$221.34\pm 4.12$	$219.66\pm 2.68$	$487.59\pm 8.34$	$486.92\pm 9.00$	$484.03\pm 8.39$	$238.73\pm 5.13$	$241.84\pm 5.38$	$237.98\pm 5.68$	$215.67\pm 3.25$	$217.78\pm 2.70$	$214.64\pm 2.69$
	MEDAE (s)	$180.65\pm 15.02$	$179.68\pm 38.10$	$178.74\pm 24.13$	$481.10\pm 7.02$	$479.32\pm 8.16$	$476.80\pm 8.67$	$242.39\pm 16.82$	$242.41\pm 24.63$	$237.62\pm 17.12$	$163.75\pm 14.74$	$156.45\pm 28.25$	$160.99\pm 17.41$
Retained	MAE (s)	$51.53\pm 1.11$	$54.22\pm 0.44$	$48.64\pm 1.10$	$47.00\pm 0.32$	$47.76\pm 0.26$	$44.31\pm 0.26$	$49.90\pm 0.67$	$50.72\pm 0.55$	$47.26\pm 0.65$	$50.75\pm 0.78$	$51.55\pm 0.62$	$48.14\pm 0.76$
	MEDAE (s)	$32.70\pm 0.88$	$35.31\pm 0.81$	$30.36\pm 0.90$	$31.13\pm 0.17$	$31.92\pm 0.30$	$28.95\pm 0.16$	$31.92\pm 0.28$	$32.75\pm 0.44$	$29.62\pm 0.30$	$32.06\pm 0.08$	$33.02\pm 0.27$	$29.90\pm 0.32$

In this table, *desc*, *fgp* and *desc+fgp* refer to the input features used by each regressor. *desc* means descriptors, *fgp* means fingerprints and *desc+fgp* means that both descriptors and fingerprints have been used

**Table 3:** MAE and MEDAE results weighted CatBoost regressors with large overall weights for non-retained molecules (1/2, 1/1 and 2/1)

Features	Metric	CB 1/2			CB 1/1			CB 2/1		
		desc	fgp	desc+fgp	desc	fgp	desc+fgp	desc	fgp	desc+fgp
All	MAE (s)	$57.78\pm 1.04$	$58.37\pm 0.82$	$55.34\pm 1.02$	$59.02\pm 1.11$	$59.52\pm 0.87$	$56.67\pm 0.89$	$60.90\pm 1.17$	$61.03\pm 0.96$	$58.49\pm 1.16$
	MEDAE (s)	$34.40\pm 0.44$	$35.02\pm 0.54$	$32.39\pm 0.69$	$35.62\pm 0.74$	$36.04\pm 0.61$	$33.71\pm 0.61$	$37.46\pm 0.66$	$37.48\pm 0.75$	$35.49\pm 0.96$
Non-retained	MAE (s)	$168.46\pm 8.31$	$169.78\pm 7.59$	$167.70\pm 8.14$	$166.46\pm 8.14$	$166.81\pm 7.80$	$165.52\pm 7.85$	$164.59\pm 7.98$	$165.19\pm 7.51$	$162.96\pm 7.96$
	MEDAE (s)	$16.12\pm 2.20$	$15.99\pm 2.05$	$15.34\pm 2.65$	$12.32\pm 2.20$	$10.93\pm 1.42$	$11.48\pm 1.94$	$9.59\pm 1.30$	$9.10\pm 1.11$	$9.49\pm 1.32$
Retained	MAE (s)	$54.86\pm 1.14$	$55.43\pm 0.87$	$52.38\pm 1.08$	$56.19\pm 1.15$	$56.69\pm 0.90$	$53.80\pm 0.94$	$58.16\pm 1.25$	$58.28\pm 1.04$	$55.74\pm 1.24$
	MEDAE (s)	$34.71\pm 0.41$	$35.38\pm 0.53$	$32.70\pm 0.63$	$36.00\pm 0.65$	$36.43\pm 0.57$	$34.06\pm 0.57$	$37.88\pm 0.65$	$37.93\pm 0.72$	$35.94\pm 0.91$

In this table, *desc*, *fgp* and *desc+fgp* refer to the input features used by each regressor. *desc* means descriptors, *fgp* means fingerprints and *desc+fgp* means that both descriptors and fingerprints have been used

Regarding the other models, they can be sorted from lower to higher errors as follows: DKL, XGBoost, and lightGBM and CatBoost algorithms, which have similar MAE. It is worth noting that DKL obtains similar results to those reported in [24] ($45.6 \pm 2.4$ and $40.8\pm 2.4\;s$ using fingerprints for all molecules and retained molecules, respectively) and [26] ($39.87\;s$ for retained molecules only).

The differences in the regressors’ performance originate from the prediction of the RTs for the retained molecules since the MAE for non-retained molecules is quite similar for all models.

### Computing the projection between chromatographic methods

#### Comparison of meta-learned GP models

**Fig. 6:**
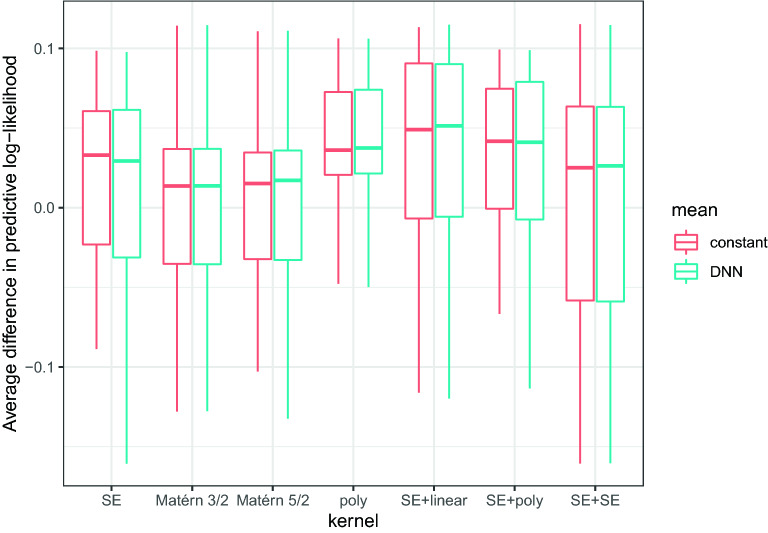
Averaged differences in predictive marginal log-likelihood $\Delta \mathcal {L}_{\text {avg}}$ for different combinations of means and kernel functions. *SE* refers to a squared exponential kernel and *poly* to a polynomial kernel of degree 4

Figure 6 shows the averaged differences in predictive marginal log-likelihood $\Delta \mathcal {L}_{\text {avg}}$ for different combinations of means and kernel functions. Since the median of all models is $>0$, the meta-learning provides some advantage with respect to directly fitting a GP to the target-task data. Using a flexible mean function parametrized by a DNN does not seem to offer any advantage compared to the simpler constant mean. Regarding the influence of kernels, although there is no clear winner, the combination of a squared exponential kernel and a linear kernel (which has the largest median $\Delta \mathcal {L}_{\text {avg}}$), and the polynomial kernel of degree 4 (which has the lowest variability) stand out. Since having a low variability is particularly important in the context of training from few points, we shall use a GP parametrized with a constant mean, and a polynomial kernel of degree 4. That is

$$\begin{aligned} f(x) \sim \mathcal {GP}\left( c, (xx' + \gamma )^4\right) . \end{aligned}$$

#### Performance of the projection function

**Fig. 7:**
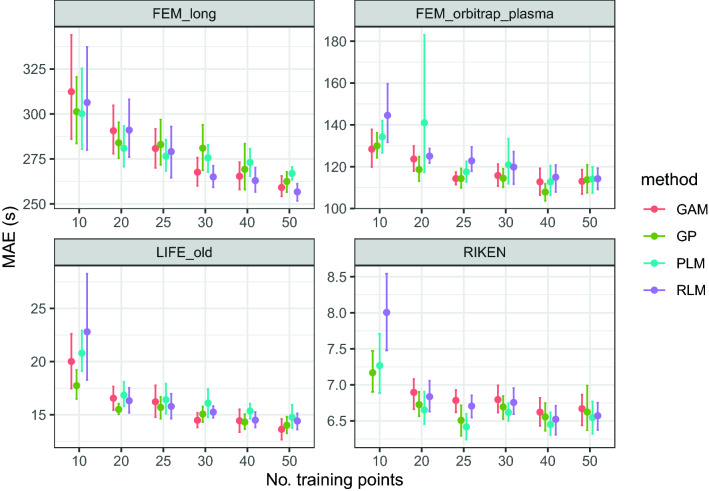
MAE for projections between the predicted RTs and different CMs when using different projection models. GP refers to a GP with constant mean and polynomial kernel of degree 4, GAM refers to monotonic Generalized Additive Models [8], RLM refers to robust polynomial regression of order 4 [21], and PLM refers to piecewise polynomial regression [24]. The GAM value for RIKEN and 10 training points is a large outlier and hence it is not shown

**Fig. 8:**
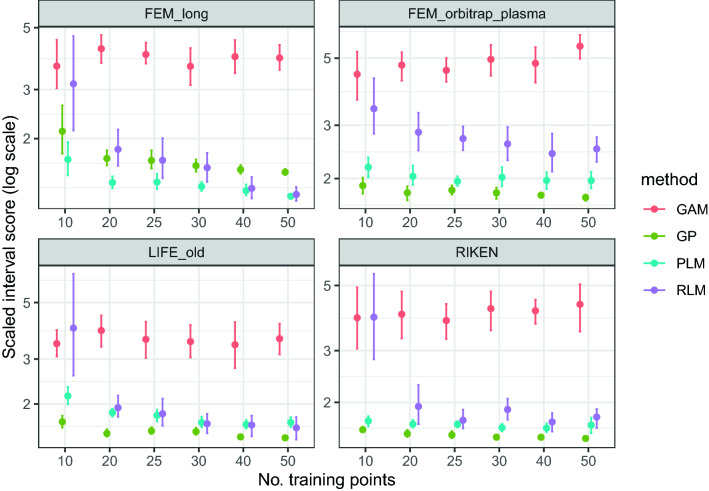
Scaled interval score (the lower, the better) for projections between the predicted RTs and different CMs when using different projection models. See Fig. 7 for the meaning of the acronyms

Figures 7 and 8 show the MAE and scaled interval scores for the projections to four CMs from the PredRet database when using different models. MEDAE results show a similar behaviour to those obtained with MAE, and hence are shown in Fig. S4 of Additional file 1. Table 4 shows these three metrics and the median relative error (in %) for the meta-learned GP. Regarding the accuracy of the model (MAE and MEDAE), all methods perform similarly. However, GPs consistently rank among the two best results for most combinations of Chromatographic Method (CM) and number of training points. Figure 7 is particularly revealing since all methods but GPs show some large fluctuation (note the large error bars) for 10 or 20 training points, which suggests that they are more sensitive to the presence of outliers.

**Table 4:** Scaled interval score, Median relative error (in %), MAE and MEDAE for projections between the predicted RTs and different CMs

CM	Metric\No. training points	10	20	25	30	40	50
FEM long	Scaled interval score	$2.27\pm 1.03$	$1.70\pm 0.19$	$1.68\pm 0.21$	$1.60\pm 0.15$	$1.55\pm 0.09$	$1.51\pm 0.06$
	Median relative error (%)	$21.64\pm 5.32$	$17.62\pm 2.19$	$17.77\pm 2.86$	$16.14\pm 1.40$	$16.76\pm 1.90$	$16.98\pm 1.36$
	MAE (s)	$301.28\pm 31.53$	$283.98\pm 17.02$	$283.02\pm 20.50$	$281.01\pm 23.22$	$269.25\pm 21.48$	$262.52\pm 9.47$
	MEDAE (s)	$237.42\pm 27.99$	$223.17\pm 11.71$	$219.34\pm 17.12$	$218.66\pm 21.25$	$210.91\pm 18.16$	$202.64\pm 9.31$
Fem orb.	Scaled interval score	$1.90\pm 0.21$	$1.80\pm 0.16$	$1.83\pm 0.10$	$1.79\pm 0.13$	$1.76\pm 0.06$	$1.73\pm 0.06$
	Median relative error (%)	$14.04\pm 1.32$	$12.73\pm 2.12$	$12.18\pm 0.86$	$11.67\pm 1.27$	$11.13\pm 0.88$	$11.76\pm 1.35$
	MAE (s)	$129.99\pm 10.55$	$118.65\pm 10.36$	$114.31\pm 7.85$	$114.45\pm 8.02$	$107.89\pm 6.63$	$113.77\pm 10.90$
	MEDAE (s)	$90.68\pm 12.43$	$74.3\pm 11.86$	$74.01\pm 12.39$	$70.64\pm 6.62$	$64.80\pm 9.54$	$66.2\pm 8.99$
LIFE old	Scaled interval score	$1.70\pm 0.15$	$1.53\pm 0.08$	$1.56\pm 0.08$	$1.55\pm 0.08$	$1.48\pm 0.05$	$1.47\pm 0.03$
	Median relative error (%)	$11.46\pm 1.93$	$9.97\pm 0.82$	$10.18\pm 1.00$	$9.88\pm 0.66$	$9.22\pm 0.61$	$9.26\pm 0.53$
	MAE (s)	$17.74\pm 2.31$	$15.50\pm 0.91$	$15.69\pm 1.74$	$15.07\pm 1.26$	$14.32\pm 1.19$	$14.01\pm 1.35$
	MEDAE (s)	$14.18\pm 2.65$	$12.01\pm 1.66$	$12.70\pm 2.15$	$11.31\pm 1.53$	$10.71\pm 1.57$	$9.93\pm 1.43$
RIKEN	Scaled interval score	$1.61\pm 0.05$	$1.56\pm 0.07$	$1.55\pm 0.07$	$1.52\pm 0.03$	$1.52\pm 0.02$	$1.50\pm 0.02$
	Median relative error (%)	$6.64\pm 0.75$	$6.13\pm 0.40$	$5.85\pm 0.31$	$5.81\pm 0.28$	$5.79\pm 0.31$	$5.81\pm 0.33$
	MAE (s)	$7.16\pm 0.50$	$6.72\pm 0.29$	$6.50\pm 0.34$	$6.69\pm 0.26$	$6.55\pm 0.32$	$6.62\pm 0.52$
	MEDAE (s)	$5.47\pm 0.65$	$4.91\pm 0.33$	$4.64\pm 0.33$	$4.80\pm 0.29$	$4.71\pm 0.37$	$4.86\pm 0.54$

Reported median relative errors for 50 training points in [21] were 17%, 14%, 10% and 8% for FEM long, FEM orbitrap plasma (Fem orb. in the table), LIFE old and RIKEN, respectively

Regarding the scaled interval scores, piecewise liner regression and meta-learned GPs show a better overall performance than the other methods, specially when compared to GAMs. Meta-learned GPs have the best performance in three of four CMs, while piecewise linear regression performs better in one of four.

**Fig. 9:**
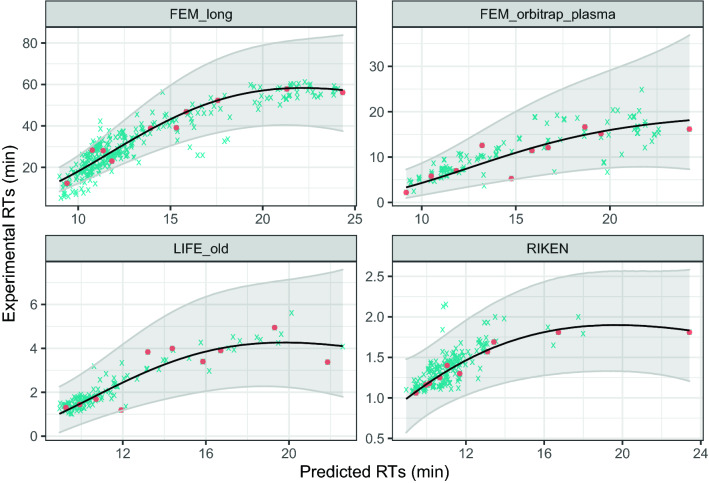
Projections from the predicted RTs to four different CMs from the PredRet database using the proposed meta-learning approach. Red points and blue crosses indicate train and test points respectively. The black line is the predictive mean of the GP while the grayed region indicates the predictive 95% interval

An illustrative example of the projection function built using just 10 training points is shown in Fig. 9.

#### Ranking candidate annotations with the projection function

Table 5 shows the percentage of the results where, using the Retention Time (RT) projection function, the true molecule was ranked among the top three candidates for those queries with more than three candidates. A comparison with the baseline values when only mass information is used (shown between parentheses in Table 5) reveals that the use of Retention Time (RT) information always improves ranking accuracy. Reported results in [21] for 50 training points were 66.7%, 67.9%, 69.7% and 71.9% for FEM long, FEM orbitrap plasma, LIFE old and RIKEN, respectively. Considering the standard error, when using 50 annotated molecules our DNN+meta-learning approach outperforms [21] in the FEM long system, while it has lower performance in the LIFE old system. The DNN+meta-learning approach reaches a global mean of $70\%$ for 50 annotated molecules. The number of training points affects both the ranking accuracy and its variability (standard errors). When using as few as 10 training points, the global performance decreases to $68\%$.

**Table 5:** Percentage of results where the true molecule was ranked among the top three candidates using the meta-learning method

CM No. training points	10	20	30	40	50
FEM long	$71.07\pm 5.11$	$72.75\pm 4.40$	$74.42\pm 5.29$	$75.27\pm 2.25$	$76.10\pm 2.92$
$$(59.57 \pm 6.73)$$					
FEM orb.	$64.96\pm 10.96$	$67.13\pm 10.04$	$65.25\pm 8.41$	$63.06\pm 9.07$	$67.04\pm 9.40$
$$(53.57\pm 7.66)$$					
LIFE old	$62.99\pm 5.21$	$61.38\pm 5.13$	$60.50\pm 6.68$	$58.57\pm 4.83$	$59.86\pm 3.36$
$$(51.44\pm 7.26)$$					
RIKEN	$72.89\pm 8.59$	$73.21\pm 4.37$	$74.44\pm 4.17$	$77.61\pm 4.04$	$75.73\pm 3.81$
$$(53.62\pm 5.77)$$					

Reported percentages in [21] were 66.7%, 67.9%, 69.7% and 71.9% for FEM long, FEM orbitrap plasma (Fem orb. in the table), LIFE old and RIKEN, respectively. Values between parentheses under the name of the Chromatographic Method (CM) represent the baseline performance when only mass information is used

## Discussion and conclusions

In this paper we have trained several state-of-the-art machine learning regressors to predict small molecules Retention Time (RT) using the 80,038 experimental RTs from the SMRT dataset. The regressors included DNNs, DKL, XGBoost, lightGBM, CatBoost, and a blending approach. The models were trained using only molecular descriptors, only fingerprints, and both types of features simultaneously. Descriptors and fingerprints were generated with the alvaDesc software. Furthermore, we have proposed a meta-learning approach to learn projection functions between different CMs from a few training points.

### Retention time prediction

Deep learning models, regardless the input features used for training, clearly outperform the other models. When using fingerprints, the DNN achieves a MAE of $39.2 \pm 1.2\; s$ when considering all molecules, and a MAE of $34.0 \pm 0.9\; s$ when considering retained molecules only; the previous top performing models achieved a MAE of $45.6 \pm 0.4\;s$ [24] on all molecules, and $39.87\;s$ when only using retained molecules [26]. This suggests that DNNs are better suited for Retention Time (RT) prediction than other models. Note that the DKL models, which should also exploit the benefits of DNNs, also achieve similar results to previously top-performing models, although they do not reach the performance of DNN. This may imply that the use of recent techniques intended for improving the generalization capabilities of DNNs (e.g.  warm-restarts and SWA) were key for their performance.

Although meta-models are expected to improve the base-regressors’ performance, the blender built using all regressors has similar performance to that obtained by DNNs (see Fig. 5). To achieve an improvement, the base-estimators of the blender should have similar performance and be as diverse as possible, providing complementary information to be exploited by the meta-regressor. In our blender, the predictions of the meta-regressor are mostly influenced by the DNNs, since they have the best performance. The fact that the blender cannot improve the predictions of the DNNs implies that their predictions are almost the same. Indeed, the predictions of the three different DNNs are highly correlated (e.g., the correlation between the fingerprints’ DNN and the descriptors’ DNN is $0.972 \pm 0.003$). Since the fingerprints’ DNN has similar performance and can be trained much faster, we can conclude that the use of blending has not provided any value for Retention Time (RT) prediction.

Figure 5 shows that models that did not employ Bayesian search (lightGBM and CatBoost) perform worse, which suggests the usefulness of this procedure. These were also the models that benefited from using both descriptors and fingerprints; in the other models using both types of features together had a performance similar to using only the descriptors. In the literature there are both works reporting that fingerprints outperform molecular descriptors (e.g., [21]) and works claiming just the opposite (e.g., [24]). Our results slightly favor the usage of fingerprints, although it cannot be ruled out that the best type of feature depends on the machine learning regressor used.

Regarding the experiments where the weights of the non-retained molecules were varied within the CatBoost regressor, Fig. 5 shows that increasing the importance of these molecules (large weights) yields worse MAE results for the retained molecules. As expected, large weights yield some improvement in the performance of the non-retained molecules (see Fig. 5). However, the large values of MAE for the non-retained molecules indicate that the regressors are not able to reliable distinguish non-retained molecules from retained ones. This also explains why the usage of different weighted CatBoosters did not have the expected impact on the blender: it was expected that the blender would match the performance of the best regressor for non-retained molecules. However, this has not been observed probably because the regressors fail to identify non-retained molecules and they tend to predict RTs as if the molecule was retained, even if it is not. This can be confirmed by inspecting the performance of the classifier trained to predict if a molecule will be retained or not (see "Preprocessing of descriptors and fingerprints" Section). Although the classifier has large specificity ($0.9953 \pm 0.0005$), precision and recall are low ($0.74 \pm 0.03$ and $0.512 \pm 0.016$, respectively), which highlights the difficulty in properly identifying non-retained molecules.

### Meta-learning-based projections

The experiments suggest that the method to project the predicted RTs to a specific Chromatographic Method (CM) is able to provide proper projections using as little as 10 or 20 training points. In this range of training points, the accuracy of the meta-learned-GP shows similar or slightly better MAE and MEDAE than other state-of-the-art methods (Fig. 7). Regarding the prediction intervals, it has the best performance in three of the four CMs (Fig. 8).

Being able to train the projection model from a few training points is key for real world applications, since it avoids the need to identify a large number of molecules. Note that in this small-data regime, the predictions are mainly driven by the prior learned during meta-learning. This can be seen by looking at the upper confidence interval for the FEM long Chromatographic Method (CM) in Fig. 9, which seems larger than needed. With more training points, the GP is flexible enough to reduce uncertainty around the training points, adjusting to the actual dispersion of the Chromatographic Method (CM), as shown by the trend for the FEM long system in Fig. 8. Remarkably, and although the scaled interval scores tend to decrease with the number of training points, they are quite stable for the other systems. The ability of GPs of generating credible prediction intervals for the projections can be used to obtain probabilistic scores for the putative annotations, as shown in "Ranking candidate annotations with the projection function" Section.

Table 5 shows that, when using 50 training points, our projection method ranks the correct identity among the top three candidates in $70\%$ of the cases, at a similar level than other projection methods [21]. When decreasing the number of training points to just 10 samples, the percentage is $68\%$, while with 30 is $69\%$. This shows that meta-learning enables the creation of projection functions from just a few known metabolites. However, Table 5 also reveals large standard errors, which suggest that the projection functions are highly dependent on the training inputs.

An accurate predictive model and a projection function that can be learned from as few as 10 identified metabolites permit building a tool to support metabolite annotation following the scheme presented in Fig. 2. We intend to integrate such a tool into CEU Mass Mediator [5], a metabolite annotation platform that has 332,665 metabolites in its database, of which approximately 250,000 have no RT information in the SMRT dataset. When RTs are available, it will only be necessary to use the projection function to map the experimental Retention Time (RT)s of the database to the Retention Time (RT) of the Chromatographic Method (CM) of a given experiment. When no Retention Time (RT) is available in the database, it will also be necessary to predict it using the best model achieved in this work (the DNN trained with fingerprints). The user of CEU Mass Mediator will only need to provide (1) the experimental RTs of the known molecules, whose identity should also be specified (by indicating their PubChem ID, InChI Key or similar), and (2) both the m/z and experimental RTs of the molecules to be annotated. This information can be uploaded to the tool’s web page using text format. CEU Mass Mediator will then return the annotations compatible with the experimental data, ranked accordingly to their z-scores.

Note that the use of the CEU Mass Mediator database avoids running the DNN in real-time. On the other hand, the projection method is highly efficient thanks to the meta-learning approach: the learning of the GP prior parameters is accomplished in an offline task, and the target-task that has to be executed online to compute the posterior distributions runs in just tenths of seconds. Hence, both the predictive model and the meta-learned projection function can be integrated into the workflow of Fig. 2 with negligible computational overhead. Furthermore, that workflow could be combined with an in silico MS/MS-based annotation approach when MS/MS data is available. In that scenario, the top predicted candidates by the model could be feed into tools that match them to the experimental MS/MS data, followed by a reranking based on both RT and MS/MS predictions.

## Supplementary Information

**Additional file 1.** Additional details on the experiments. Section S3 provides a brief description of the kernels tested with the DKL methods and the meta-learned GPs. Section S2 illustrates the training and validation procedures for all Retention Time (RT) regressors. Section S3 describes an additional experiment studying the influence of the number of meta-tasks in the performance of meta-learned GPs. Section S3 shows the performance of the machine learning models used to predict RTs using the MEDAE metric. Section S4 shows the performance of different projection methods using the MEDAE metric.

## Data Availability

The software supporting the conclusions of this article is available in the *constantino-garcia/cmmrt* Github repository (https://github.com/constantino-garcia/cmmrt).“The sofware is distributed as a Python 3 package (platform independent) and also provides a Makefile for running most important actions (i.e, installing dependencies, train and validate regressors, and train and validate projections). Furthermore, the fingerprints and descriptors generated with alvaDesc are automatically downloaded when running the software.
