# Supplementary material for: Probabilistic metabolite annotation using retention time prediction and meta-learned projections
Source: J Cheminform. 2022 Jun 7;14:33. doi: 10.1186/s13321-022-00613-8 (PMC9172150; doi:10.1186/s13321-022-00613-8)
Supplement: Supplementary file 1 — Additional file 1. Additional details on the experiments. Section S3 provides a brief description of the kernels tested with the DKL methods and the meta-learned GPs. Section S2 illustrates the training and validation procedures for all Retention Time (RT) regressors. Section S3 describes an additional experiment studying the influence of the number of meta-tasks in the performance of meta-learned GPs. Section S3 shows the performance of the machine learning models used to predict RTs using the MEDAE metric. Section S4 shows the performance of different projection methods using the MEDAE metric. [file 13321_2022_613_MOESM1_ESM.pdf]

Supporting Information: Probabilistic metabolite annotation using retention time prediction and meta-learned projections

Constantino A. García[1], Alberto Gil-de-la-Fuente[1,2], Coral Barbas[2], and Abraham Otero[1,2]

[1] Department of Information Technology, Escuela Politécnica Superior, Universidad San Pablo CEU, Spain
[2] Centre for Metabolomics and Bioanalysis (CEMBIO), Facultad de Farmacia, Universidad San Pablo CEU, Spain
constantino.garciama@ceu.es

S1 Kernels

Table S1 shows the kernels tested with the Deep Kernel Learning (DKL) regressor and to create meta-learned Gaussian Processes (GPs).

Kernel	$k_{\boldsymbol{\theta}}(x, x')$	$\boldsymbol{\theta}$	Description
Squared exponential (SE)	$\sigma^2 \exp\left(-\frac{(x-x')^2}{2\ell^2}\right)$	$\boldsymbol{\theta} = [\sigma, \ell]$	σ controls the variance of the kernel and ℓ is the length-scale parameter (determining the typical length that we should move in the input space to observe a significant change in the function).
Matérn	$\sigma^2 \frac{2^{1-\nu}}{\Gamma(\nu)} \left(\sqrt{2\nu} d\right)^{\nu} K_{\nu}\left(\sqrt{2\nu} d\right)$ with $d = \frac{(x-x')^2}{2\ell^2}$ and where K_ν is the modified Bessel function	$\boldsymbol{\theta} = [\sigma, \ell]$	Parameters have the same meaning as with SE kernel. Note that ν is not part of $\boldsymbol{\theta}$ and hence it is not optimized.
Linear	$\sigma^2 x \cdot x'$	$\boldsymbol{\theta} = [\sigma]$	σ controls the variance of the kernel.
Polynomial	$(x \cdot x' + c)^d$	$\boldsymbol{\theta} = [c]$	c is an offset parameter. Note that d is not part of $\boldsymbol{\theta}$ and hence it is not optimized.
Spectral mixture	$\sum_{q=1}^{Q} w_q e^{-2\pi^2\tau^2 v_q} \cos\left(2\pi\tau\mu_q\right)$ with $\tau = x - x'$	$\boldsymbol{\theta} = [\boldsymbol{w}, \boldsymbol{\mu}, \boldsymbol{v}]$ with $\boldsymbol{w} = [w_q]_{q=1}^{Q}$, $\boldsymbol{\mu} = [\mu_q]_{q=1}^{Q}$, $\boldsymbol{v} = [v_q]_{q=1}^{Q}$	The kernel is a mixture of Q Gaussians with means $\boldsymbol{\mu}$, variances $\boldsymbol{v}$ and weights $\boldsymbol{w}$.

Table S1: Kernels tested with DKL and meta-learned GPs.

S2 Training and validation procedures for retention times prediction

Figure S1 summarizes the training and validation procedures for all tested retention times predictors, with a focus on the blending approach.

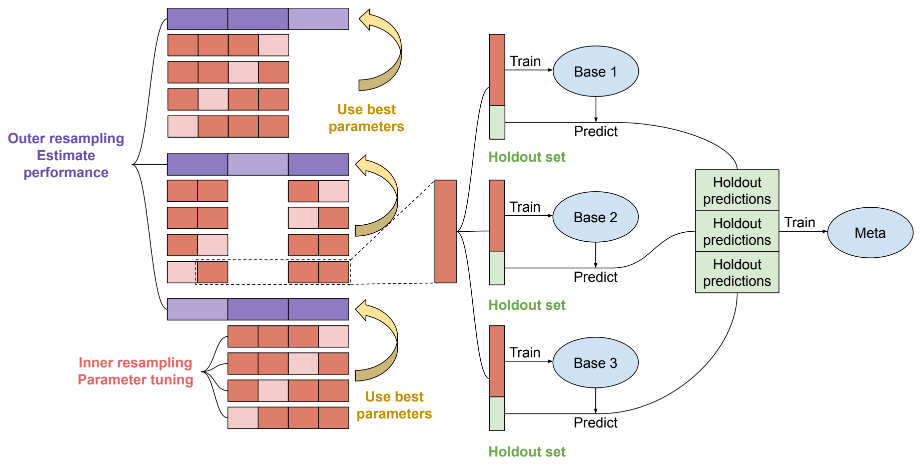

Figure S1: Outline of the training and validation procedures. Outer cross-validation estimates performance by averaging the results on the light boxes. Each dark box is used to create an inner cross-validation loop which is then employed to tune the model parameters. Again, dark boxes are used to train and light boxes are used to test parameter settings. The figure uses 3 and 4 folds for the outer and inner loops for simplicity. The training procedure for the blender is outlined. A holdout set (green box) is created using a small subset of the training set. The remaining training data (red box) is used to train the base-regressors, which then make predictions on the holdout set. Predictions are merged together to create a new dataset, which is used to train the meta-regressor.

S3 Influence of the number of meta-tasks on meta-learned Gaussian Processes

We have evaluated how the number of meta-tasks may influence the performance of the best model from Section 3.2.2. To that end, we used a similar experimental setup to that described in Section 3.2.1, but with a few adaptations.The MEDian Absolute Error (MEDAE) was used as metric for the experiment, since it is a robust statistic. For a given target Chromatographic Method (CM), the number of meta-tasks was increased from 1 to 23 (since PredRet database contains 24 different CMs) in steps of 4 or 5. To increase the number of meta-tasks, we considered adding new ones randomly. However, this resulted in very noisy curves without any clear relation between the number of meta-tasks and MEDAE. A possible explanation for this is that, when adding new tasks, some may be similar to the target CM (and the prior benefits from learning from them), while others may be dissimilar (hence not helping the target CM in learning a good prior).

To tackle the issue, we first computed a similarity score between all tasks. A GP with constant mean and polynomial kernel was fit to each CM (no meta-learning involved) and the euclidean distance between the fitted parameters was computed. We then tested two approaches to increase the number of meta-tasks. In the first approach, we started with the meta-task most similar to

the target-task and added new meta-tasks in decreasing order of similarity. In the second approach, we started with the most dissimilar meta-task and added meta-tasks with increasing similarity. After meta-learning on the current active set of meta-tasks, performance on the target-tasks was evaluated 10 times with different target training data and target test data. We focused on the low data regime using only 10 target training points.

Results in Figure S2 show the influence of the number of meta-tasks on the MEDAE for several target CMs. The two colors correspond to the strategy followed to add new meta-tasks. When meta-learning starts with the most similar meta-task and adds new meta-tasks in decreasing order of similarity, the MEDAE can be deemed as almost flat (red curve). Hence, meta-learning can learn from a single meta-task provided that it is similar enough to the target-task. On the other hand, when meta-learning starts with the most dissimilar meta-task and adds meta-tasks with increasing similarity, MEDAE decreases with the number of meta-tasks except for the FEM orbitrap plasma CM, where the pattern is almost flat. This may be because FEM orbitrap plasma can be considered as an "average CM". This is illustrated in Figure 1 of the main manuscript. FEM orbitrap plasma is an "average CM" in the sense that its projection curve lies in the middle of the projection space. (On the other hand, both FEM long and LIFE old show projection curves that lie above and below most projection curves, respectively.) Hence, even when meta-learning from the most dissimilar task, the resulting prior is close to the optimal one for FEM orbitrap plasma, thus showing good performance.

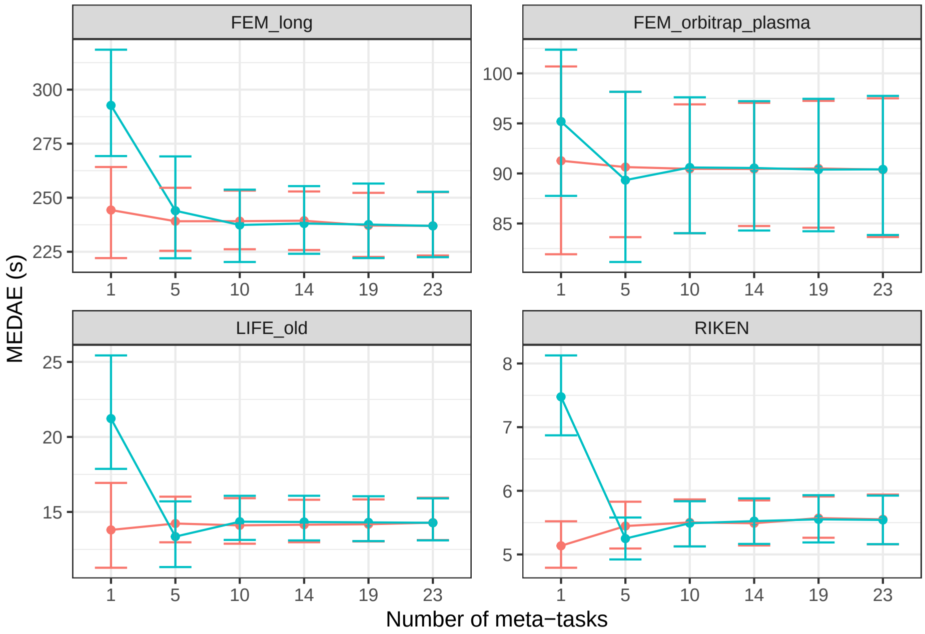

Figure S2: Influence of the number of meta-tasks on the MEDAE for several target CMs. The two colors correspond to the strategy followed to add new meta-tasks. Red color starts with the most similar task and adds meta-tasks in decreasing order of similarity. Blue color starts with the most dissimilar task and adds meta-tasks in increasing order of similarity.

Figure S2 shows that the similarity between meta and target tasks is more relevant than the number of meta-tasks. However, since the target task is not known in advance, in practice it would be best to use as many meta-tasks as possible to ensure that at least one of them is similar to the target-task.

S4 MEDAE in retention time prediction

The performance of the models developed for Retention Time (RT) prediction is summarised in Figure 5 of the main document, which shows the Mean Absolute Error (MAE) of all machine learning algorithms (see Section 4.1). As a complement to Figure 5, Figure S3 shows the MEDAE results for the same algorithms. Note that the behavior is qualitatively the same as in Figure 5, and hence Figure S3 was not included in the main document.

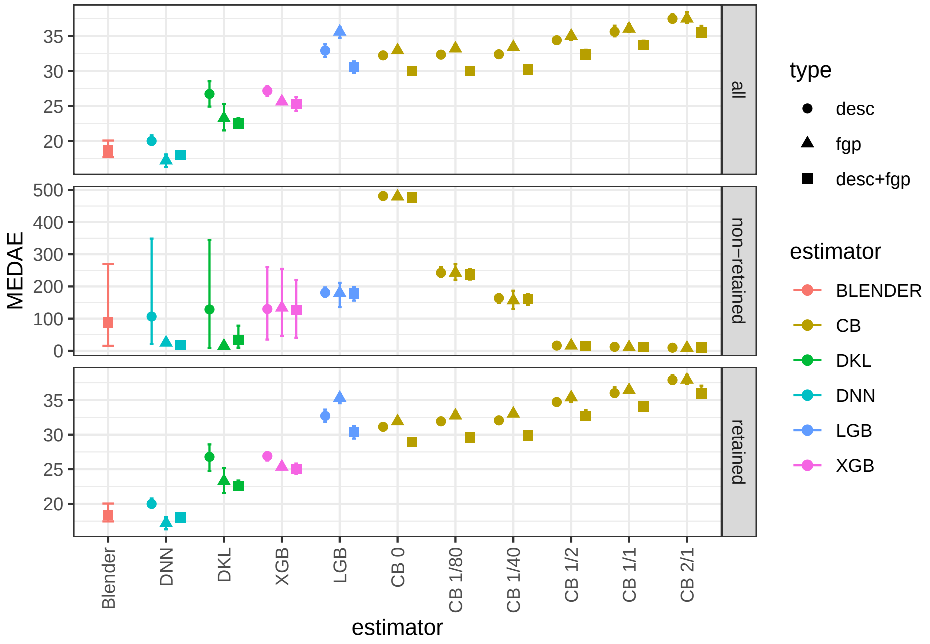

Figure S3: MEDAE results in seconds. See Figure 5 in the main document for an explanation of the legend.

S5 MEDAE for projections between CMs

Figure 7 in the main document shows the MAE for different projection methods when mapping predicted RTs to different CMs (see Section 4.2.2). Figure S4 shows the MEDAE for the same projection methods. Note that the behavior is qualitatively the same as in Figure 7, and hence Figure S4 was not included in the main document.

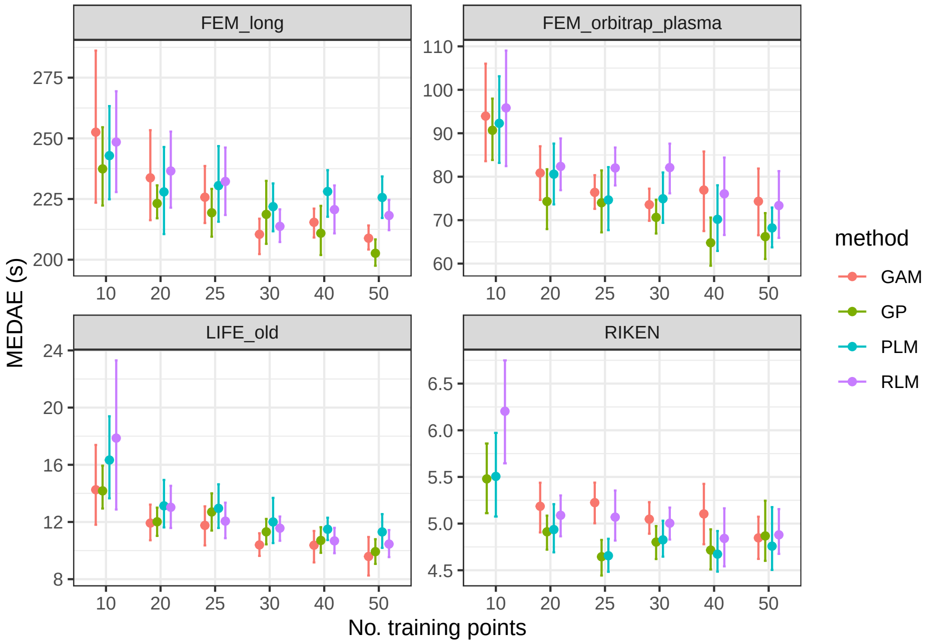

Figure S4: MEDAE for projections between the predicted RTs and different CMs when using different projection models. See Figure 7 in the main document for the meaning of the acronyms. The GAM value for RIKEN and 10 training points is a large outlier and hence it is not shown.
